# Leveraging Geospatial Data Science to Uncover Novel Environmental Predictors of Cardiovascular Disease∗

**DOI:** 10.1016/j.jacadv.2023.100371

**Published:** 2023-06-07

**Authors:** Sadeer Al-Kindi

**Affiliations:** School of Medicine, Case Western Reserve University and the Harrington Heart and Vascular Institute, University Hospitals, Cleveland, Ohio, USA

**Keywords:** cardiovascular disease, environment, prediction

**Figure:**
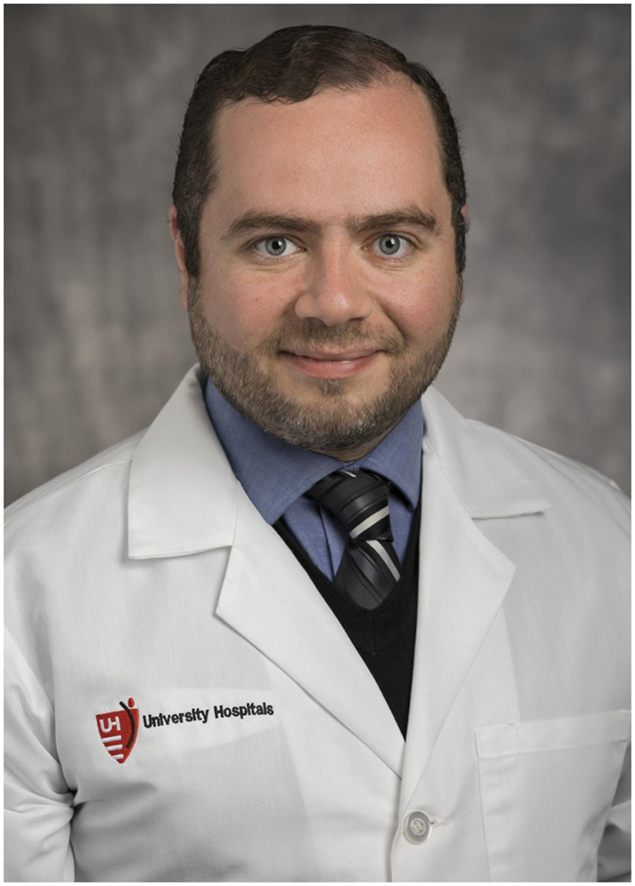

“Data are not information, information is not knowledge, knowledge is not understanding, understanding is not wisdom,”

—Clifford Stoll

Cardiovascular disease (CVD) remains a major cause of morbidity and mortality globally. While traditional risk factors such as hypertension, high cholesterol, smoking, and diabetes have been well-studied, recent research has shown that the environment also plays a significant role in the development and progression of CVD.^1^^,^^2^ Environmental factors such as air pollution, exposure to toxic chemicals, noise pollution, and climate change have been linked to increased risk of CVD.^3^ Air pollution alone is estimated to cause approximately 9 million premature deaths globally every year, with majority being due to CVD.^4^ Additionally, high and low temperatures have been associated with increased mortality risk, mainly due to CVD.^3^ Investigating the impact of meteorologic factors and air quality on health outcomes is becoming increasingly important as we tackle the climate crisis.^3^ While considerable evidence exists for air pollution (particularly particulate matter) effects on human health, the impact of other environmental factors, such as temperature and temperature changes, climate, wind, and other meteorologic factors on CVD risk are less well understood. Given the large number of temporally and spatially varying environmental exposures that an individual experiences, understanding their impact on human health outcomes requires complex geospatial analyses.

In this issue of *JACC: Advances*, Vishram-Nielsen et al^5^ perform an elegant analysis of a large cohort of individuals in Canada. The investigators leveraged data from 24 million individuals in Canada with >1.7 million CVD hospitalizations over 10-year period (2007-2017), which they linked to 17 weather factors (including air pressure, wind speed, humidity, ultraviolet radiation) and air pollutants (particulate matter, SO_2_, NO_2_, CO). To investigate the association between environmental factors and CVD events, the authors used Besag-York-Mollié model,^6^ a Bayesian statistical method commonly used in spatial epidemiology and disease mapping to analyze the distribution and incidence of diseases across geographic areas. The model accounts for both spatial and nonspatial sources of variation in disease occurrence, allowing for a more accurate assessment of the relative risk of disease in different locations. The authors built age-specific spatiotemporal models to explore the association between environmental variables and the incidence of CVD hospital admissions (namely, heart failure, myocardial infarction, and stroke), allowing for interaction between the terms. The study team identified age-specific environmental markers of CVD risk that also varied by the specific event type (heart failure, myocardial infarction, or stroke). Interestingly, the study shows that there are complex interactions among the environmental factors and that the association between environmental factors and CVD was stronger among older patients. For example, air pollutants and wind speed were more strongly associated with events among young individuals, while low temperature, high/low wind speed, high/low pressure, wet/dryness, and air pollutants were associated with CVD events among older individuals. Models incorporating environmental factors outperformed models that included spatial and temporal factors alone.

The second aim of the study was to develop a CVD event prediction tool based on environmental factors. Using the factors that were defined in the association study, the developed prediction model had near-perfect spatial estimation of all CVD events, and explained 49% (R^2^ = 0.49), 49% (R^2^ = 0.49), and 22% (R^2^ = 0.22) of the spatiotemporal variation in heart failure, myocardial infarction, and stroke, respectively.

Prior studies have combined environmental factors to identify association with health outcomes in a Bayesian framework,^7^^,^^8^ but as the authors correctly report, these studies are limited by inclusion of small number of exposures, without investigating the interactions between pollutants and weather effects, thus limiting interpretability. The current study extends prior work by highlighting the age-environment interaction, unraveling novel relationships that may improve predictive accuracy. Additionally, allowing for interactions between the environmental factors, the study shows complex intersection between air quality/weather variables with CVD among the different age groups.

Accordingly, the current study provides a detailed analysis of the relationship between weather conditions and air pollution with CVD risk, showing that approximately 50% of spatiotemporal variation in myocardial infarction and heart failure are explained by environmental factors. It further highlights the limitation of prior environmental studies that incorporate single pollutants, without accounting for co-pollutants and concomitant meteorologic changes.

As the authors correctly identified, there are limitations to this study and further research is needed to clarify some of the observed findings. For example, the use of administrative data sets has limited accuracy which may introduce bias into the study findings. Additionally, there is no characterization of comorbidities or traditional risk factors that can vary spatially (and to a certain degree temporally) and may introduce bias to the model. Characterization of the social environment is also essential to unravel the impact of environmental susceptibility.^9^^,^^10^ Lastly, it is also important to investigate the applicability of such a model to other regions to ensure external generalizability.

This study has implications for clinical practice, policy making, and resource allocation. In clinical practice, identifying comprehensive environmental early warnings (such as the one developed in this study) for susceptible patients (eg, those who have prior CVD events) may help modify patients behaviors such as staying indoor during these events.^11^ Additionally, identifying the relevant factors for CVD events can guide policy development to identify the sources of such pollutants, and introduce new standards to curb them. Finally, given the significant variation in CVD hospitalizations, the developed prediction models can help allocate additional health care resources (eg, repurposing hospital rooms to CVD events, recruitment of travel physicians and nurses, broadening telehealth utilization) that can facilitate health care delivery during the periods of adverse environmental risk.

As medical applications of computational approaches continue to evolve, I envision data-driven multiomics platforms (Figure 1) that integrate clinical data, genomics, epigenomics, radiologic factors (radiomics), social factors (sociomics), environmental factors (environmics), and wearable technology to characterize exposomic (exposure) determinant of health. Such platforms will allow us to understand the complex paradigms of ‘nature vs nurture’ in a detailed fashion, and to develop CVD-specific spatiotemporal neighborhood fingerprints to allow early warning systems and early intervention. Such systems can enable understanding of the residual environmental risk for CVD events and facilitate innovation in CVD risk reduction strategies as we adapt to climate change.

**Figure 1:**
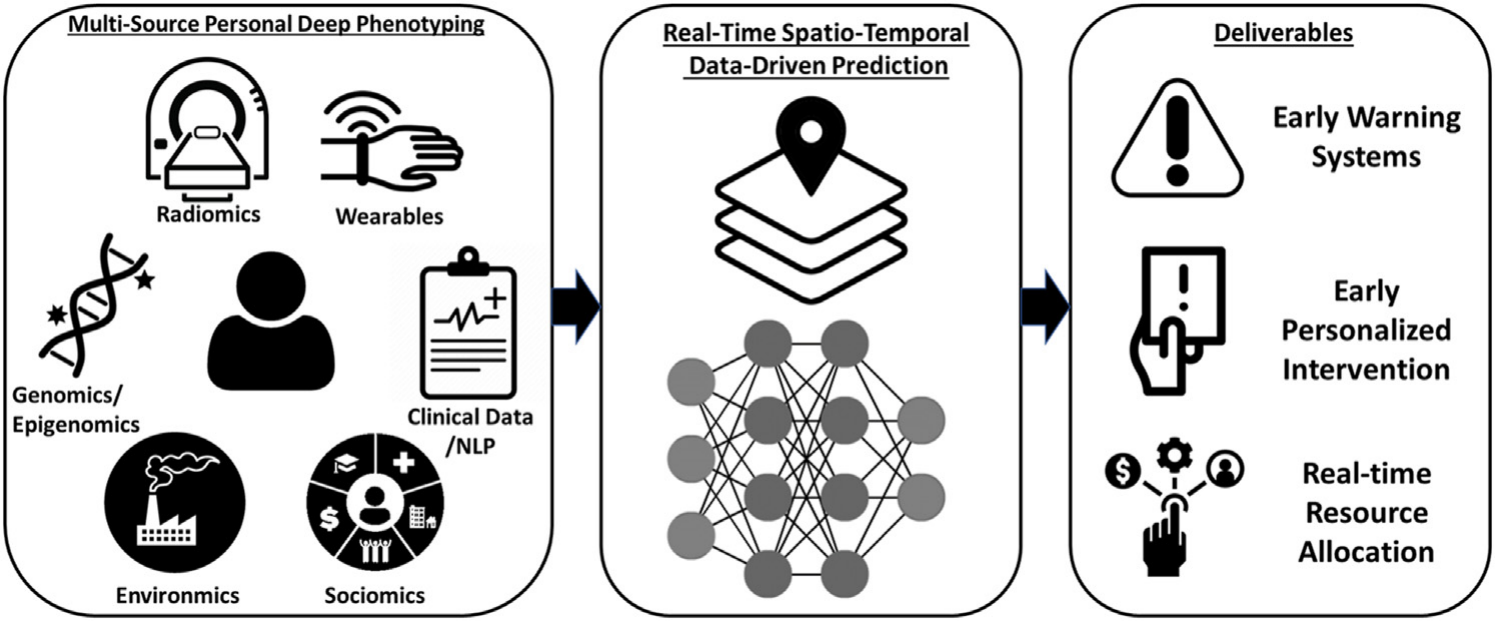
**Conceptual Framework for Future Real-Time Risk Prediction for Cardiovascular Disease Events** Data sources include radiomics, wearable devices, genomics/epigenomics, clinical data with natural language processing (NLP), environmental and social data. Data are integrated using spatiotemporally enabled computational models, allowing for real-time early warning systems for populations, personalized early intervention, and real-time health care resource allocation.

## Funding support and author disclosures

This work was partly funded by the 10.13039/100006545National Institute on Minority Health and Health Disparities Award #P50MD017351. The author has reported that he has no relationships relevant to the contents of this paper to disclose.

## References

[1] Al-Kindi S.G., Brook R.D., Biswal S., Rajagopalan S. (2020). Environmental determinants of cardiovascular disease: lessons learned from air pollution. Nat Rev Cardiol.

[2] Rajagopalan S., Al-Kindi S.G., Brook R.D. (2018). Air pollution and cardiovascular disease: JACC state-of-the-art review. J Am Coll Cardiol.

[3] Khraishah H., Alahmad B., Ostergard R.L. (2022). Climate change and cardiovascular disease: implications for global health. Nat Rev Cardiol.

[4] Landrigan P.J., Fuller R., Acosta N.J. (2018). The Lancet Commission on pollution and health. Lancet.

[5] Vishram-Nielsen J.K.K., Mueller B., Ross H.J. (2023). Association between the incidence of hospitalizations for acute cardiovascular events, weather, and air pollution. JACC: Adv.

[6] Besag J., York J., Mollié A. (1991). Bayesian image restoration, with two applications in spatial statistics. Ann Inst Stat Math.

[7] Silva G.L., Dean C.B., Niyonsenga T., Vanasse A. (2008). Hierarchical Bayesian spatiotemporal analysis of revascularization odds using smoothing splines. Stat Med.

[8] Shin H.H., Stieb D., Burnett R., Takahara G., Jessiman B. (2012). Tracking national and regional spatial-temporal mortality risk associated with NO2 concentrations in Canada: a Bayesian hierarchical two-level model. Risk Anal Int J.

[9] Bevan G., Pandey A., Griggs S. (2022). Neighborhood-level social vulnerability and prevalence of cardiovascular risk factors and coronary heart disease. Curr Probl Cardiol.

[10] Motairek I., Lee E.K., Janus S. (2022). Historical neighborhood redlining and contemporary cardiometabolic risk. J Am Coll Cardiol.

[11] Kelly F.J., Fuller G.W., Walton H.A., Fussell J.C. (2012). Monitoring air pollution: use of early warning systems for public health. Respirology.

